# Motivations of wheelchair curling athletes to participate in sport: a qualitative research

**DOI:** 10.3389/fpsyg.2025.1623063

**Published:** 2025-11-19

**Authors:** Sonay Serpil Daşkesen, Deniz Bedir, Ekrem Levent İlhan, Tuğba Demir Onur, Heather Mair

**Affiliations:** 1 Department of Physical Education and Sports Teaching, Faculty of Sport Sciences, Atatürk University, Erzurum, Türkiye; 2 Faculty of Sport Sciences, Erzurum Technical University, Erzurum, Türkiye; 3 Faculty of Sport Sciences, Gazi University, Ankara, Türkiye; 4 Department of Recreation and Leisure Studies, Faculty of Health, University of Waterloo, Waterloo, ON, Canada

**Keywords:** wheelchair curling, disabled athletes, paralympic sports, motivation, self-determination theory

## Abstract

**Introduction:**

Self-Determination Theory (SDT) is a holistic approach that provides a comprehensive framework for understanding individuals’ intrinsic and extrinsic motivations, analyzing the nature of motivation and its relationship with psychological development. This study employs the SDT framework to examine the motivational processes of wheelchair curling athletes.

**Methods:**

This qualitative study involved eleven wheelchair curling athletes. Data were collected through a personal information form and semi-structured interviews. The collected data were analyzed using thematic analysis, and themes related to athletes’ motivational experiences were identified.

**Results:**

The findings revealed that the motivation of wheelchair curling athletes to participate in sport is influenced by both intrinsic and extrinsic factors. Intrinsic motivation, nourished by physical, emotional, and social sources, strengthens athletes’ participation in curling, enhances their enjoyment, and reinforces their commitment to the sport. In contrast, extrinsic motivation is shaped by elements such as external regulation, introjected regulation, identified regulation, and integrated regulation. However, a motivation mainly stems from lack of resources and organizational deficiencies, which negatively affect athletes’ participation in and continuity of the curling sport.

**Discussion:**

The study emphasizes the importance of individual and structural factors influencing the motivation of wheelchair curling athletes. Strengthening organizational support and improving resource accessibility can enhance athletes’ intrinsic motivation, thereby promoting participation and sustainability in adapted sports.

## 1 Introduction

The sport of curling, which dates back at least 500 years, is defined as “chess played on ice” and has a long history (Hattori et al., 2016). It first emerged in Scotland (Willoughby and Kostuk, 2004), was introduced to Canada by Scottish immigrants in the 18th and 19th centuries and its popularity spread from there (Mott and Allardyce, 1989). In 1924, it gained an international identity at the Winter Olympics and has been a full medal sport since 1998 (World Curling, 2025). This development diversified with the addition of wheelchair curling to the Paralympic program at the 2006 Torino Paralympic Winter Games (Giovanis and Margari, 2015; IPC, 2024) and mixed doubles curling becasme a medal sport in 2018 (IPC, 2024). Today, the appeal is widespread. It is estimated that approximately 2 million people play curling (Ağduman and Bedir, 2023), and World Curling (the international body, governing the sport) has 73 member nations (World Curling, 2025). Further, it has been described as the world’s fastest growing winter sport (Baldwin, 2018).

Curling, stands out with its unique rules and strategy-based structure. In its most common iteration, two teams of four players (Gao et al., 2024) compete on an ice surface 4 feet wide and 146 feet long. This surface is called a “sheet” with a concentric circular area with diameters of 12, 8, and 4 feet at each end. This circular area is called a “house” or “rings”. At the end of the game, the team with the stones (also called rocks) closest to the center of the house wins points (Kostuk and Willoughby, 2006). The game’s dynamic nature is not limited to the placement of the stones, as the shooting process and sweeping technique are also strategically important. The throwing process is completed when the thrower moves by pushing off from the starting block (called a “hack”), glides for about 10 m, and then releases the stone (Berry et al., 2013; Kivi and Auld, 2012). When a player throws the stone, two teammates use special sweeping techniques, which clear the ice, heat the surface, and help control the stone’s speed and direction (Bradley, 2009; Marmo et al., 2006).

Wheelchair curling is an adaptation for disabled individuals (e.g., people with physical disabilities such as spinal cord injuries, cerebral palsy, multiple sclerosis, or lower limb amputation). In wheelchair curling, basic rules are preserved, but some elements, such as sweeping are removed are removed [Gao et al., 2024; World Curling Federation (WCF), 2024]. The absence of sweeping changes the game’s strategy, making the thrower’s job more difficult and making it necessary to release the stone with the correct speed, angle, and force. In this case, reaching the target depends entirely on the thrower’s technical skills.

This adaptation both increases the technical difficulty and significantly reduces the possibility of injury (i.e., by eliminating the risks of slipping or falling during sweeping; Bradley, 2009). In addition, the absence of sweeping for individuals with disabilities, whose participation in physical activity is often limited due to limitations in motor skills, increases accessibility, facilitating participation, and increasing opportunities for social interaction (Wang et al., 2022). Indeed, wheelchair curling is an important adaptation, which allows individuals to participate, thereby promoting physical activity and positively effecting an individual’s quality of life through increased social interaction. Although wheelchair curling offers important opportunities for disabled individuals in terms of both physical activity and social participation, the motivational factors behind participation in this sport have not been sufficiently investigated. Thus, it is important to identify intrinsic and extrinsic motivational sources for athletes to continue their participation in curling. Further, understanding motivational processes can contribute to developing strategies to increase athletes’ long-term commitment as well as the benefits they receive from participation.

Motivation is an essential element that mobilizes the individual in a specific direction. In the context of sport, motivation plays a critical role in ensuring the continuity of an individual’s participation and can improve their performance (Deci and Ryan, 1985). Understanding motivation can strengthen the factors that encourage athletes to continue in sportand help address barriers that may cause them to quit (Tekkurşun-Demir and İlhan, 2020). Although there are various studies on the motivation of disabled and non-disabled individuals’ participation in sport (Tekkurşun-Demir and İlhan, 2019; Moradi et al., 2020; Bojkowski, 2022; Kontro et al., 2022; Alp and Alpdoğan, 2023), there is no such study of wheelchair curling.

Recent systematic reviews and meta-analyses further highlight the complex motivational landscape of athletes with disabilities and adaptive sport participants. For instance, Aitchison et al. (2022) synthesized qualitative studies and showed that participation in adaptive sports enhances autonomy, competence, and relatedness, key needs identified in self-determination theory. Similarly, Isidoro-Cabañas et al. (2023) reported in their meta-analysis that adaptive sport engagement improves physical and mental quality of life, with intrinsic enjoyment and social connection acting as principal motivators. Global evidence compiled by Ginis et al. (2021) demonstrated that accessible environments and supportive social networks are critical facilitators of sustained physical activity for individuals with disabilities. Focusing specifically on barriers, Elipe-Lorenzo et al. (2025) identified transportation, equipment availability, and limited promotion as major obstacles to mainstream sport participation for people with disabilities. Complementing these findings, Liu et al.’s (2024) systematic review highlighted the pivotal role of coach–athlete relationships in nurturing need satisfaction and internalization of extrinsic motives. Collectively, these recent syntheses underscore the relevance of SDT for understanding how adaptive sport participants internalize external regulations and sustain long-term involvement, thereby reinforcing the need to explore motivational processes in wheelchair curling athletes.

Nonetheless, participation in a particular sport is effected by different motivational sources (Weinberg et al., 2000). The lack of research with wheelchair curling athletes makes it challenging to adequately understand motivational processes in this particular context and could prevent the development of strategies to encourage more participation in sport. This situation creates a significant research gap in terms of understanding the effects of motivation on athlete performance andretention, especially for Paralympic and accessible sport more broadly.

### 1.1 Theoretical framework

This study was used self-determination theory (SDT) to understand and analyze the motivational processes of wheelchair curlers. Self-determination theory is an approach that provides a holistic framework for understanding individuals’ intrinsic and extrinsic motivations and analyses the nature of motivation and its relationship to an individual’s psychological development (Ryan and Deci, 2000). SDT was founded by Deci and Ryan in the 1980s, and in the following years, subsequent studies refined the theory into a more comprehensive and clearly defined framework. SDT suggests that individuals are motivated when their three innate psychological needs—competence, autonomy, and relatedness—are met and that the satisfaction of these needs supports a person’s development. In other words, individuals are more motivated and can sustain their development when they can make their own choices, have the opportunity to develop their competencies, and establish meaningful connections with their environment (Ryan and Deci, 2000). Self-determination theory examines the types of motivation that direct individuals’ behaviors in relation to the following three main categories.

#### 1.1.1 Intrinsic motivation

It refers to the situation in which an individual performs an activity because he/she enjoys it, the activity improves himself/herself, or it provides personal satisfaction. The basis of intrinsic motivation is that an individual performs an activity only for enjoyment, development, and satisfaction, regardless of external rewards (Ryan and Deci, 2000). This type of motivation contributes to an individual exhibiting high performance, increasing creativity, encouraging deep learning, and enhancing satisfaction (Ryan and Deci, 2020).

#### 1.1.2 Extrinsic motivation

It refers to a situation where an individual is driven by external factors, such as earning rewards, gaining social approval, or avoiding punishment. In this type of motivation, what matters is not so much the activity itself but the results it produces (Ryan and Deci, 2000). According to SDT, extrinsic motivation is not a one-dimensional phenomenon but is assessed on a continuum that shows the extent to which an individual has internalized their behavior. This continuum starts with the least autonomous form, external regulation, passes through the stages of introjected regulation and identified regulation, and reaches the most autonomous form, integrated regulation. In external regulation, behavior is entirely motivated by reward or avoidance of punishment, while in introjected regulation, the individual imposes pressure on themselves and behavior is sustained by internal pressures, such as shame or guilt. Identified regulation is related to consciously accepting the value and importance of behavior; integrated regulation, on the other hand, refers to behavior becoming consistent with the individual’s identity and integrating with their philosophy of life. Therefore, external motivation is not merely an instrumental gain but a dynamic process that can integrate with the individual’s values and identity (Ryan and Connell, 1989; Ryan et al., 1992; Ryan and Deci, 2000).

#### 1.1.3 Amotivation

It refers to the situation in which the individual has no intrinsic or extrinsic motivation and, therefore, cannot make sense of the activity (Ryan and Deci, 2020). An individual’s perception that he/she cannot control the outcome of an action has a determining effect on behavior and motivation. When an individual believes that an action he/she performs will not lead to a direct result, this may lead to a decrease in the capacity to act and a decrease in motivation. In other words, when an individual thinks that his/her efforts will not change the outcome, his/her motivation decreases, and he/she shows reluctance to exhibit relevant behaviors (Deci and Ryan, 2012).

Various researchers have examined SDT in the context of sports and have established this theory as an important framework for understanding the motivational processes of athletes (Spray et al., 2006; Lourenço et al., 2022). For example, within the SDT framework the motivational processes of athletes are understood through the dynamics between intrinsic and extrinsic motivation (Rodrigues et al., 2019). Regarding intrinsic motivation, athletes’ participation in sports is generally associated with factors such as personal fulfillment, development, and excitement of competition. Explorations of extrinsic motivation indicate athletes are influenced by rewards, social recognition, and coaching pressure (Trigueros et al., 2019). As noted above, no efforts to apply the SDT framework to understand the specific context of wheelchair curling athletes exist. Therefore, our study aimed to utilize SDT to understand the motivational processes of wheelchair curling athletes and sought to reveal factors shaping the athletes’ continuity in the sport.

It should be noted that there are only 18 licensed wheelchair curling athletes in Turkey, and this is an indication that participation is quite limited and as such, understanding the motivational processes of current athletes would be an important step in encouraging more individuals to participate. Further, as is noted above, SDT illustrates how developing strategies that can strengthen intrinsic motivation may be critical to increasing athletes’ long-term commitment to any sport. Indeed, making sports more attractive for individuals with disabilities and creating appropriate incentive mechanisms can improve their participation and performance. In this respect, the framework of self-determination theory offer a valuable tool for understanding the motivational processes of athletes and developing practices, strategies, approaches, or regulations that support participation in sports.

## 2 Method

### 2.1 Research design

This research was designed using qualitative research methods, in particular phenomenological design. Phenomenological design aims to investigate how individuals experience a phenomenon or phenomena and the meanings they give to these experiences (Johnson and Christensen, 2019). Given its focus on experience and meaning, phenomenology, shaped by SDT, is an appropriate approach for understanding and interpreting the motivations of wheelchair curling athletes.

### 2.2 Participants

Criterion sampling method was used in the study as it allows the researcher to select individuals or cases with specific characteristics related to the phenomenon they wish to examine (Büyüköztürk et al., 2018). Accordingly, participation in the 2023–2024 Wheelchair Curling National Team trials was set as the criterion. İn total, 11 wheelchair curling athletes (8 men, 3 women) participated in the study. Considering that there are only 18 licensed wheelchair curling athletes in Turkey, 11 athletes represents a significant portion of the population. Although a relatively small sample size is typical for phenomenological research, a sample of 11 athletes also represents approximately 60% of the licensed wheelchair curling athletes in Turkey. While this provides insights close to the population level, caution is still required when generalizing to all athletes at the national or international level. Data saturation was achieved as the data collected generated recurring themes and no new information emerged. Specifically, only a minor subcode emerged after analysis of the ninth interview, and no new codes were added as a result of the tenth and eleventh interviews, confirming data saturation. Therefore, it was accepted that the current sample size was sufficient to fulfill the purpose of the study. The athletes’ ages ranged from 22 to 53 (*x̄* = 40 ± 11.0) and their sporting careers ranged from 2 to 22 years (*x̄* = 7.36 ± 6.25). Pseudonyms were used to protect participants’ personal information (see Table 1).

**Table 1:** Participant information.

Participant	Gender	Age	Length of time in the sport	Education status	Economic status	Marital status	Onset of disability
Ahmet	Male	45	12	Bachelor’s degree	High income	Single	Acquired disabilities
Selim	Male	43	6	High school	High income	Single	Acquired disabilities
Günay	Male	53	2	Bachelor’s degree	Middle income	Married	Acquired disabilities
Büşra	Female	22	4	Bachelor’s degree	Low income	Single	Acquired disabilities
Kenan	Male	27	4	High school	Middle income	Single	Acquired disabilities
Batuhan	Male	49	3	Elementary school	Middle income	Married	Congenitally disabled
Aylin	Female	26	3	Middle school	Middle income	Single	Congenitally disabled
Hakan	Male	46	2	High school	Middle income	Married	Acquired disabilities
Burak	Male	45	22	High school	Middle income	Married	Acquired disabilities
Mehmet	Male	43	12	Associate degree	Middle income	Single	Acquired disabilities
Sevinç	Female	41	11	Associate degree	Middle income	Single	Acquired disabilities

### 2.3 Data collection tool

Personal information forms and semi-structured interview guides were used as data collection tools. The personal information form was designed to collect information, such as gender, age, length of time in the sport, educational status, economic status, marital status, and onset of disability of the athletes. The nine questions in the semi-structured interview guide were designed to generate an in-depth understanding of the motivations of wheelchair curling athletes to participate in sports. Specifically, the guide was designed using insights from relevant extant literature and insights from the research team, which included two expert academicians and the lead researcher (who conducted the interviews). Sample questions that guided the discussion with wheelchair curling athletes are as follows:

- What are the aspects of participating in sport that motivate you the most? (Intrinsic Motivation)
- How do external factors (family, coaches, rewards, social expectations, etc.) motivate you to participate in sport? (Extrinsic Motivation)
- What are the factors that force you to participate in curling or reduce your motivation? (Amotivation)

### 2.4 Collection of data

Interviews with the athletes were conducted face-to-face in the curling hall on 28 May, 2024. The participants were given detailed information about the confidentiality and anonymity of the interview process and how personal data would be protected within the framework of ethical rules. The interviews were conducted in a quiet, private, and adequately lit environment, which was organized in a way that would not disturb the participants in terms of temperature and noise, each interview lasted between 25 and 30 min. The audio-recorded interviews were transcribed, and the data set was read several times.

### 2.5 Researcher reflexivity

Throughout the study, the research team remained attentive to how their professional backgrounds in sport sciences and experience with athletes with disabilities could shape both data collection and interpretation. We approached the interviews and thematic analysis with an awareness of these potential influences, deliberately discussing our assumptions and perspectives during team meetings, and coding sessions. Ongoing, reflective conversations and careful documentation of analytic decisions were used to ensure that findings were grounded in participants’ own words and experiences rather than in researcher expectations. While no formal bracketing protocol was implemented at the outset, this continuous reflexive stance served to minimize bias and enhance the transparency and trustworthiness of the results.

### 2.6 Data analysis

In the study, the data obtained from the interviews were analyzed using the thematic analysis method. The analysis of the data was carried out by following an inductive approach based on the stages identified by Braun and Clarke (2006) as “the researcher’s familiarity with the data, creating the first codes, searching for themes, reviewing the themes, defining and naming the themes and preparing the report” (p. 87). Firstly, codes were created, and then these codes were transferred to the Microsoft Excel 2024 program. During the coding process, the interviewee to whom each code belonged was indicated, and the interviewee’s statements that were remarkable and explanatory in terms of the research were also included on the sheet. These statements were considered important because they were individual narratives and provided insights that explained motivational processes and overlapped with the SDT theory. For example, while some participants emphasized their sense of autonomy, others mentioned their relationship with teammates (relatedness) or their perception of sporting competence. Such insights allowed us to understand the motivational experiences of athletes in more depth, especially when assessed in light of the SDT framework (Deci and Ryan, 2000). Codes, categories, and themes were organized systematically by opening different sheets in Excel. This structure ensured that the analysis process was more consistent and understandable and contributed to clearly presenting the research findings.

### 2.7 Validity—reliability

In order to ensure the validity and reliability of the research, various strategies were followed for credibility, transferability, consistency, and confirmability (Lincoln and Guba, 1985). In order to ensure the reliability of the research, a “checking meeting” was conducted with the athletes, during which the findings were shared and the athletes confirmed that the results accurately reflected their experiences. All analyses were reported clearly and in detail by the principle of verifiability. In order to ensure consistency, the interview coding keys and interview transcripts were independently analyzed by the researchers and two experts. Necessary arrangements were made by discussing the issues involving “consensus” and “disagreement.” The reliability calculation of the study was made using the reliability formula proposed by Miles and Huberman (1994). This formula calculates the reliability percentage as “Percentage of Agreement = (Agreement)/(Agreement + Disagreement) × 100.” Miles and Huberman (1994) suggest that the reliability percentage should be above 70%. In this study, the percentage of agreement was calculated as 94%, which reveals the study’s high reliability.

#### 2.7.1 Ethics committee approval

Before starting the study, the ethics committee report dated April 19, 2024 and numbered E-70400699-000-2400142335 was obtained from Ataturk University Ethics Commission. Written informed consent was obtained from the individual(s) for the publication of any potentially identifiable images or data included in this article.

### 2.8 Limitations

Athletes were included in the study without discriminating between men and women. There may be differences between genders. This situation can be shown as the limitation of the research.

## 3 Findings

In this study, three themes (intrinsic motivation, extrinsic motivation, and amotivation) and nine categories (physical sources of motivation, emotional sources of motivation, social sources of motivation, external regulation, introjected regulation, identified regulation, integrated regulation, lack of resources, and organizational deficiencies) emerged. Tables 2–4 present the emerging themes, categories, and codes.

**Table 2:** Intrinsic motivation.

Theme	Category	Code	Participant
Intrinsic motivation	Physical sources of motivation	Feeling vigorous	Ahmet, Günay, Burak, Mehmet, Sevinç
		Strengthening the upper limb muscles	Selim, Aylin, Burak, Sevinç
	Emotional sources of motivation	Having self-confidence	Selim, Kenan, Batuhan, Burak, Sevinç
		Happy, free, peaceful	Ahmet, Aylin, Hakan, Mehmet, Sevinç
		Feeling special because few people do it	Selim, Büşra, Burak
		Love to do sports	Ahmet, Günay, Batuhan, Aylin, Hakan, Burak
	Social sources of motivation	Entering the community	Selim, Günay, Büşra, Kenan, Batuhan, Aylin, Hakan, Burak, Mehmet, Sevinç
		To meet local and foreign athletes with the same disability group	Selim, Büşra, Kenan, Batuhan, Aylin
		Popularising curling	Sevinç

**Table 3:** Extrinsic motivation.

Theme	Category	Code	Participant
Extrinsic motivation	External regulation	On recommendation	Kenan, Hakan
	Introjected regulation	Prove themselves	Kenan, Mehmet, Sevinç
	Identified regulation	Win medals and lift trophies	Ahmet, Selim, Büşra, Hakan, Burak, Mehmet, Sevinç
		Selection for the national team	Günay, Büşra, Kenan, Batuhan, Aylin, Burak, Mehmet, Sevinç
	Integrated regulation	Being a role model	Günay

**Table 4:** Amotivation.

Theme	Category	Code	Participant
Amotivation	Lack of resources	Lack of auxiliary personnel	Hakan
		Lack of equipment	Mehmet
	Organizational deficiencies	Accessibility and availability	Ahmet, Selim, Günay, Büşra, Hakan, Burak, Mehmet, Sevinç
		Lack of information and promotion activities with state support	Selim, Kenan, Batuhan, Aylin, Mehmet

### 3.1 Intrinsic motivation

Three categories have emerged in the theme of intrinsic motivation: physical sources of motivation, emotional sources of motivation, and social sources of motivation. In terms of gender distribution, the vast majority of codes in these themes were expressed by both female and male athletes. However, it is noteworthy that the code “Popularising curling” was expressed by only one female athlete. The fact that female athletes in Turkey still face visibility and representation issues may be a possible explanation for this. Therefore, it can be considered that female athletes may interpret sport not only in the context of their individual motivations, but also in terms of being role models for others and contributing to society.

#### 3.1.1 Physical sources of motivation

The findings revealed that physical development was a significant factor in increasing wheelchair curlers’ motivation to participate in sports. Athletes viewed sports not only as a means of maintaining their physical health but also as an opportunity to strengthen and improve their performance. For example, Günay emphasized the contribution of sports to physical vitality, saying, *“Going out and exercising instead of staying at home keeps me physically fitter.”* Similarly, Burak stated, *“Exercising is very beneficial for our physical development. Curling is one of these sports. It increases blood circulation. It keeps you more vigorous and fit, and I can say that it strengthens and develops your arms,”* these insights indicate that physical benefits are a meaningful source of motivation. These statements could also demonstrate that meeting athletes’ competence needs is a key element in enhancing their motivation.

#### 3.1.2 Emotional sources of motivation

The most frequently cited source of intrinsic motivation by wheelchair curlers was emotional factors. Athletes emphasized that participation in this sport gives them a sense of self-confidence, peace, and distinction. For example, Burak stated*, “Curling increases your self-confidence. As people’s perspectives of us change, we develop emotionally.”* This insight indicates that sports strengthen self-esteem not only through performance but also through the transformation of social perceptions. Ahmet emphasized the contribution of sports to autonomy and emotional well-being, saying, *“I feel more peaceful and happy, especially on the ice. I feel more free. I feel completely isolated from disability.”* Selim’s statement, *“I feel very different… It does not matter how cold we are. Because we overcome this, I see myself as different from those who do not participate in this sport,”* demonstrates that overcoming challenges gives individuals a sense of distinction and a special identity.

These findings indicate that increased self-confidence and strengthened self-esteem fulfill the need for competence; feelings of peace and freedom fulfill the need for autonomy; and changes in social perceptions fulfill the need for relatedness. Therefore, it can be concluded that participation in curling meets athletes’ basic psychological needs and thereby enhances their intrinsic motivation.

#### 3.1.3 Social sources of motivation

The second most frequently mentioned category in the theme of intrinsic motivation was social sources of motivation. Athletes stated that participating in curling offered them not only physical exercise but also significant contributions in terms of community involvement, developing social relationships, and a sense of belonging.

Mehmet’s statement highlights the importance of interacting with different social circles through curling: *“I think participating in curling provides great social development. As you can see, you meet people from different provinces, with different characters. You are in the same environment. We breathe the same air. When you go abroad for your sport, you meet new people there. Whatever your discipline, there are many benefits to socialising.”* This statement shows that participation in sport strengthens social bonds and meets athletes’ need for relatedness.

Hakan’s words point to the role of sport in increasing social participation: *“Socialising is one of the main reasons that motivated me to participate in this sport. If you have a disabled child who is interested in these types of sports, you get them out of the house. You integrate them into society. Children get out of the four walls. They socialise more. If you have disabled children, you should direct them toward these types of sports. Children become more socialised.”* This insight reveals that ensuring the participation of disabled individuals in society supports their need for relatedness; at the same time, it strengthens their need for autonomy by encouraging them to leave their homes and expand their social living spaces.

Sevinç’s statements highlight both the social and organizational benefits of curling: *“Making friends through curling is wonderful. Curling is not very common in our country. My greatest desire is to popularise this sport. The more disabled friends we can reach, the more teams we can form and establish a league in our own country or city, for example, we can establish a league and meet different people. But of course, curling is not suitable for every disabled group. Because it is a cold sport, not every disabled person is enthusiastic about it, which is why I want to popularise it. In other words, I want it to reach a wider audience.”* Sevinç’s words show that forming new friendships meets the need for relatedness, while the goals of promoting the sport, forming new teams, and creating a league support the athletes’ need for competence. In conclusion, these findings, emerging under the category of social sources of motivation, reveal that participation in curling nourishes athletes’ intrinsic motivation by fulfilling their needs for relatedness, autonomy, and competence.

### 3.2 Extrinsic motivation

Under the theme of extrinsic motivation, there are four categories: external regulation, introjected regulation, identified regulation, and integrated regulation (Table 3). Introjected regulation and identified regulation were expressed by both female and male athletes, whereas external regulation and integrated regulation were expressed only by male athletes. According to SDT, external regulation represents the lowest level of autonomy, and behavior is entirely driven by external rewards, punishments, or environmental pressures. The fact that female athletes did not provide responses that related to this category may suggest that they express their participation in sport with more subjective and internalised reasons rather than solely with external pressures. In contrast, Integrated Regulation represents the most autonomous form of external motivation and involves integrating sport with identity. The fact that responses by female athletes did not relate to this category suggests that opportunities to integrate sport with their identity at this level may be constrained by structural barriers (e.g., limited participation opportunities, low visibility, gender roles). These findings indicate that gender may shape the ways in which motivation is expressed.

#### 3.2.1 External regulation

The External Regulation category refers to athletes participating in sport due to external pressures or expectations, such as the suggestions or encouragement of others. This situation demonstrates that guidance from the environment is particularly influential in athletes’ decision-making processes. Indeed, Kenan expressed his views on this matter as follows: *“At first, I had reservations about taking up this sport, but a friend I knew from the wheelchair basketball team told me how enjoyable wheelchair curling was and that the club was good. Thanks to his advice, I took up the sport and am really enjoying it.”*

#### 3.2.2 Introjected regulation

Some athletes stated that they were influenced by internal pressures such as seeking approval for their participation or avoiding feelings of guilt and this relates to the Introjected Regulation category. For example, Kenan reflected an internalised sense of responsibility, emphasizing that the motivation to make his family and country proud was very important. Kenan: *“I want to represent my country well. I did not come here just to play; I want to make my family proud.”*

#### 3.2.3 Identified regulation

The identified regulation category helps us understand how athletes may internalise success and social status, not only as external expectations but also as personally valuable and meaningful goals. In this context, the desire to be selected for the national team stands out as one of the most powerful factors shaping athletes’ motivation. Indeed, Mehmet expressed this situation with the following words: *“The external factor that motivates me the most is my goal of being selected for the national team. My primary goal is to join the national team squad and then participate in major events such as world championships or world qualifiers in the disciplines we will compete in. We have a mixed tournament ahead of us, and I am working to represent my country in the best possible way at this tournament. Doing our best and winning a medal, regardless of its colour, would be a great achievement for me. But my biggest dream is to participate in the Olympics and experience that atmosphere.”*

#### 3.2.4 Integrated regulation

The integrated regulation category refers to situations where participation in sport is fully aligned with the athlete’s values and self-perception. In this context, Günay’s motivation demonstrates that sport has become an integral part of his identity. His emphasis on setting an example for young people and his own children, encouraging them to participate in sport, and even including disabled individuals in his neighbourhood in sport, reveals that sport is not just an activity for him, but a way of life that reflects his personal values and social responsibilities. Günay: *“I want to be a role model for young people and my own children. This motivates me. When we come here to play sports, it’s not appropriate for our young people to sit in coffee shops. They should come too and set an example. For example, I got my own children started in sports. One of them may make it to the national team. I also do archery, I find disabled people in the neighbourhood and bring them along. I have disabled archers too.”*

### 3.3 Amotivation

Under the theme of lack of motivation, two categories emerged: lack of resources and organizational deficiencies. Notably, lack of resources was only mentioned by male participants. This may indicate that male athletes focus more on physical resource deficiencies that directly affect their autonomy and competence needs. In contrast, organizational deficiencies were mentioned by both female and male participants; in particular, inadequacies in accessibility, usability, and state-supported promotional activities emerged as a common obstacle for both groups. This difference suggests that gender roles may shape how participation experiences in sport are expressed, and that female athletes tend to emphasize organizational and relational dimensions rather than resources.

#### 3.3.1 Lack of resources

Two key themes stand out under the lack of resources category: lack of auxiliary personnel and lack of equipment. When assessed in terms of SDT’s fundamental psychological needs, these codes point to different dimensions. Lack of auxiliary personnel is particularly related to the obstruction of the need for autonomy. Athletes require support personnel to participate independently in training and competitions; the absence of this support limits their autonomy. Hakan expressed this situation as follows: *“Our state needs to support these types of sports. Today, there is not a single employee to take us to our car. You need to come with an escort from your home to drop us off here and take us to the hall. They could put an authorised person here. Therefore, the state should support these people, these athletes.”* On the other hand, the Lack of Equipment code is linked to the obstruction of the need for competence. The lack of necessary equipment and tools limits athletes’ opportunities to demonstrate and develop their skills. Mehmet described this situation as follows: *“We do this sport on ice. This situation makes it difficult for us. For example, we want our friends to participate. You will tell them to come, but if they do not have chairs or clothes, how will they do it here? They will come and get sick.”*

#### 3.3.2 Organizational deficiencies

Organizational deficiencies have been the most frequently cited source of demotivation among wheelchair curling athletes. An analysis of participants’ statements reveals that these deficiencies directly hinder the fulfillment of fundamental psychological needs. Aylin’s emphasis on the lack of promotion and visibility indicates that the need for relatedness is not being adequately met. The fact that curling is not sufficiently recognized and supported in society makes it difficult for athletes to gain social acceptance and develop a broader sense of belonging. Aylin expressed this situation with the following words: “*I think curling should be promoted and advertised. There is more basketball and volleyball for disabled people on television. But there is no curling. Why not? Because we started with five people. Look, we are growing slowly. It could be featured in advertisements, magazines, newspapers. We recommend it to our friends, but they do not even understand what kind of sport we are talking about. The fact that it is so little known and appreciated negatively affects my motivation.”*

The accessibility and transport issues raised by Büşra and Sevinç are particularly related to the obstruction of the need for autonomy. Being unable to reach sports facilities independently limits athletes’ capacity to implement their own decisions and move freely in the process of participating in sport. This situation leads individuals to feel dependent on external factors in terms of continuing to participate in sports and threatens long-term participation.

Büşra expressed this situation with the following words: *“Low accessibility is one of the biggest obstacles to our participation in sports. If this problem is solved, a major problem will be eliminated. Accessibility is essential in every area, from transport to lifts. Because our families also approach the issue differently. They may ask, If you do not have money, what are you doing at home?.” Our families already have jobs, so there are situations like “How can we take you there, how can we bring you back?.” If accessibility is resolved, if we can get to and from sports venues ourselves, all problems will be solved.”* Similarly, Sevinç highlighted the negative impact of transport on independent participation: *“Transport is our biggest problem. There should be a service for us. Because, for example, we go to a training session; imagine we stay here for 2 h. If we do not have our own vehicle, especially in winter, it’s very difficult for us to leave here, get on the bus, and go home. Especially when leaving the house in a wheelchair is so difficult.”*These findings show that structural barriers directly affect not only physical needs but also psychological needs; in particular, the failure to meet the need for autonomy is a fundamental factor that undermines athletes’ motivation.

Another noteworthy finding is that accessibility issues were only raised by athletes who became disabled later in life. This may stem from the fact that this group feels the loss of autonomy in their daily lives more acutely. In contrast, athletes who were born with disabilities may have internalised these structural limitations as a natural part of their lives and therefore felt no need to express them directly.

## 4 Discussion

This study examines the motivational processes of wheelchair curling athletes in the context of self-determination theory. While the existing literature focuses on general motivational factors for individuals’ sport participation (Ahmed et al., 2020; Moradi et al., 2020), research on how wheelchair curling athletes experience this process and the factors that shape their motivation is limited. Within the scope of this study, the sport participation processes of wheelchair curling athletes were examined within the framework of intrinsic and extrinsic motivation sources and inhibiting factors. The research findings were shaped around three main themes (intrinsic motivation, extrinsic motivation, and amotivation). While the data analysis confirms some of the motivational elements in the previous literature, they also provide new findings specific to wheelchair curling athletes.

### 4.1 The role of intrinsic motivation

When the intrinsic motivation themes of wheelchair curling athletes are evaluated, it is understood that the participation of these athletes is significantly affected by physical, emotional, and social factors. When the physical category was examined, it was stated that the athletes felt more vigorous and strong thanks to wheelchair curling; it provided muscle strengthening, increased physical mobility, and contributed to general health by accelerating circulation. This finding is consistent with previous studies showing that individuals with disabilities improve their physical health through sport (Wilhite and Shank, 2009; Halabchi et al., 2017; Aitchison et al., 2022). In addition, meta-analyses have revealed that physical activity positively affects cardiovascular fitness, musculoskeletal fitness, and cardiometabolic risk factors (Ginis et al., 2021). Overall, physical activity in individuals with disabilities positively affects cardiometabolic health, bone health, and obesity control in young people. For adults, physical activity also significantly reduces all-cause mortality, hypertension risk, certain types of cancer, and the incidence of type 2 diabetes (Carty et al., 2021). In this context, positive physical health outcomes are seen as an important intrinsic motivator for wheelchair curling athletes to participate in sport.

When emotional sources of motivation were examined, it became clear that wheelchair curling athletes gained self-confidence, felt happy, free, and peaceful, and saw this sport as a special experience because few people did it and loved it. The participants’ statements show that curling contributes not only to physical development but also to psychological well-being. In particular, the fact that the athletes felt a sense of freedom and independence on the ice reveals that the sport contributes to strengthening their identity. This finding supports the effect of the sense of “autonomy,” emphasized in SDT (Deci and Ryan, 1985), on the motivation to participate in sports. Engaging in the sport enhanced athletes’ sense of autonomy by fostering greater choice and control. This sense of freedom is also directly related to athletes’ self-confidence. The self-confidence reported by the athletes is in line with the findings in the literature. In the study conducted by Bačanac et al. (2014), specific psychological characteristics of disabled athletes (*n* = 12) and non-disabled athletes (*n* = 12) were compared, and it was concluded that the psychological profiles of disabled athletes were mainly similar to those of non-disabled athletes. It was also found that practicing sports positively affected the self-confidence and self-esteem of disabled athletes.

In temrs of social for intrinsic motivation, analysis of the interviews illustrated how the athletes interacted more with society, expanded their circle of friends, and strengthened their social ties through curling. In particular, meeting athletes from the same disability group and making connections at an international level enabled individuals to feel belonging to a community through sport. This finding is directly related to the need for “relatedness” emphasized in self-determination theory (Deci and Ryan, 1985), and a broader perspective, it is also in line with relationship motivation theory (RMT) also developed by Deci and Ryan (2014). Social motivation identifies the need for relatedness as one of the basic psychological needs supporting individuals’ intrinsic motivation, development, and social integration. In this context, the fact that curling athletes increase their social interactions and feel themselves as part of a community through sport contributes to meeting the need for relatedness that exists in human nature and supports the general well-being of individuals. This aligns with the literature that sport is a tool to promote social integration (Bailey, 2005; DePauw and Gavron, 2005; Valdés and Reyes, 2024).

### 4.2 Extrinsic motivation role

When examining the extrinsic motivation themes of wheelchair curling athletes, it was observed that some athletes, under the umbrella of external regulation, began participating in sports in response to guidance from family members or a close social circle. This demonstrates the decisive role of the social environment in sports participation and is consistent with findings in the literature. Indeed, Arslan et al. (2021) stated that parents and siblings are among the most important factors encouraging individuals to participate in sports. However, as Zahariadis et al. (2006) also emphasized, social orientation alone does not provide a sustainable source of motivation in the long term. The development of intrinsic motivation and personal enjoyment of sports is critical for athletes to continue participating in sports.

In the internalised regulation category, some athletes stated that internal pressures such as seeking approval or avoiding feelings of guilt had an effect on their participation in sport. For example, Kenan emphasized that the motivation to make his family and country proud is very important to him, reflecting this level of responsibility. The motive of making the family proud is a frequently observed source of internal pressure in athletes and reveals that motivation is conditioned by a sense of self-worth. The literature also supports these findings. Indeed, a qualitative study conducted with wheelchair basketball players reported that being admired and appreciated by those around them as individuals with disabilities that participate in sports was a powerful source of motivation (Sarol, 2024).

However, the negative aspects of internalised motivation are open to debate. This type of motivation can make an individual’s self-worth conditional; it carries the risk of feeling valuable when successful and worthless when unsuccessful. Research shows that self-worth efforts fuelled by internalised motivation offer limited benefits and can threaten the athlete’s psychological well-being (Tahir et al., 2024). It is stated that athletes who feel excessive internal pressure are particularly prone to burnout syndrome. Indeed, a study conducted with elite athletes found that internalised regulation significantly predicted all dimensions of athlete burnout (Tahir et al., 2024). These results indicate that while internalised motivation is a powerful driving force that encourages participation and continuity in sport, when taken to extremes, it can be a risk factor that weakens athletes’ psychological resilience.

Although winning medals, being selected for the national team, or representing one’s country may initially appear to be external motivational factors, when athletes identify these goals with their own values, their motivation rises to a defined level of regulation. Indeed, qualitative studies conducted with elite athletes have revealed that athletes place importance on “the meaning represented by the medal rather than the medal itself” (Mallett and Hanrahan, 2004). Successful athletes have stated that “A medal is proof that a person has achieved something; it is a tangible demonstration of their competence,” thereby expressing that they actually identify the medal with their own values of success and competence (Mallett and Hanrahan, 2004). In this context, winning a medal takes on meaning as a symbol of the validation of athletes’ hard work, talent, and perseverance, rather than simply obtaining a prize. Another important contribution of motivation at the defined regulation level is the balanced fulfillment of the need for social acceptance. The athlete sees the recognition they receive from society and their team as a natural result of their own efforts; this sense of acceptance also satisfies their need for relatedness in a healthy way. Thus, while experiencing feelings of belonging and appreciation, the athlete can continue to participate in sport without losing self-control of their motivation. Findings in the literature also show that defined (and integrated) motivation styles support continuity, psychological well-being, and success in sport (Mallett and Hanrahan, 2004).

As Ryan and Deci (2017) noted, at the integrated regulation level, motivation aligns with an individual’s philosophy of life and identity. However, it is noteworthy that the findings directly reflecting integrated regulation in our study were limited. This limitation suggests that athletes with disabilities may have limited opportunities to fully integrate sport participation into their identities due to structural and contextual constraints. Indeed, Shapiro and Martin (2010) emphasize that the identity development of individuals with disabilities through sport is directly related to social acceptance, accessibility, and supportive environmental conditions. Similarly, Pensgaard and Sorensen (2002) stated that achieving integrated regulation is difficult in environments where athletes’ sense of autonomy and belonging are not supported. In this context, creating more inclusive and supportive sport environments for athletes with disabilities may contribute to the integration of sport participation into individual identity.

### 4.3 Amotivation and factors hindering participation

When the theme of lack of motivation of wheelchair curling athletes is examined, it is seen that lack of resources and organizational deficiencies are among the main factors that prevent athletes from participating. One of the most concrete indicators of this situation is that there are only 18 licensed wheelchair curling athletes in Turkey. Athletes identify the most important barriers to participation as lack of appropriate equipment, insufficient support staff, lack of state-sponsored promotion, accessibility and accessibility problems, and lack of state support. Especially the fact that curling halls are not widespread throughout Turkey negatively affects the participation of physically disabled individuals living in different cities. Difficulties in transportation to training grounds are another important factor that can decrease athletes’ motivation. The inability of athletes to reach the halls on their own and the lack of auxiliary personnel to meet their various needs when they arrive make regular participation difficult and increase dependence on parental or family member assistance. This situation makes it challenging to participate individually and negatively affects the athletes’ continuity in sports. These findings are consistent with previous studies that reveal the diversity and scope of the barriers faced by individuals with disabilities in sports participation (Rimmer et al., 2004; Jaarsma et al., 2014; Fitzgerald, 2018; Elipe-Lorenzo et al., 2025). For instance, in a scoping review examining the effect of the environment on participation of children and youth with disabilities, the most common barriers identified were the physical environment, transportation, policies, and lack of support from service providers (Anaby et al., 2013). In a study of people with intellectual disabilities, the barriers experienced by participants in sports participation included lack of accessible information about programs, cost of transportation, lack of companions or supporters to facilitate participation, and discriminatory attitudes of other participants or organizations (Wright et al., 2019). Another study conducted with visually impaired individuals found that transportation is one of the most significant barriers to physical activity (Hillan et al., 2023).

Consequently, resource and organizational deficiencies can lead to failure to meet basic psychological needs (autonomy, relatedness and competence), which in turn reinforces athletes’ lack of motivation. The findings show that taking into account not only environmental and structural barriers but also individuals’ psychological needs plays a critical role in supporting disabled athletes’ participation and continuity in sport.

## 5 Conclusion and recommendations

The findings of this study show that the motivational processes of wheelchair curling athletes are multidimensional and affected by both intrinsic and extrinsic factors. Indeed, we can make the following, specific conclusions:

- In terms of intrinsic motivation, athletes’ physical, emotional, and social gains increase their participation in sports.
- Regarding extrinsic motivation, success and the desire to be represented at the national team level are strong driving forces.
- Amotivation, specifically related to issues of accessibility, lack of equipment, and organizational inadequacies, are the main factors that negatively affect the participation of athletes.

To enhance the long-term participation of individuals with disabilities in wheelchair curling and other adaptive sports, practical recommendations can be reframed as design principles rooted in self-determination theory. Sports environments should be developed to support autonomy by ensuring barrier-free facilities, reliable and affordable transportation, and flexible scheduling so athletes can make self-directed choices about when and how they participate. Providing state-supported adaptive equipment, trained auxiliary personnel, and individualized coaching can enhance perceived competence, helping athletes build skills and confidence. Inclusive media campaigns and community outreach that highlight shared experiences and success stories can strengthen relatedness, reducing stigma and increasing social recognition. Finally, multi-level social support networks (for example, such as family engagement programs, peer mentoring, and community partnerships) should be integrated to foster belonging and mutual encouragement. Implementing these SDT-aligned principles can simultaneously satisfy athletes’ needs for autonomy, competence, and relatedness, leading to stronger intrinsic motivation, higher retention, and a more inclusive adaptive sports culture. Moreover, given the indications of gendered differences in motivation observed in this study, future research should further investigate these differences in greater depth.

## Data Availability

The data presented in this study are available on request from the corresponding author. The data are not publicly available due to ethical and privacy restrictions.

## Author’s note

This study was presented as an oral presentation at the 8th International Congress of Exercise and Sport Sciences, held on April 24–26, 2025.
